# IoT MEMS: IoT-Based Paradigm for Medical Equipment Management Systems of ICUs in Light of COVID-19 Outbreak

**DOI:** 10.1109/ACCESS.2021.3069255

**Published:** 2021-03-29

**Authors:** Abdulaziz Aborujilah, Abubaker-Eseddig Fathi Mahmoud Elsebaie, Shamsul Anuar Mokhtar

**Affiliations:** Malaysian Institute of Information Technology (MIIT), University of Kuala Lumpur 117142 Kuala Lumpur 50250 Malaysia

**Keywords:** Coronavirus (COVID-19 Pandemic), medical equipment management system, MEMS, IoT, Internet of Things, total hospital information systems, THIS, and intensive care unit, ICU

## Abstract

Recently, COVID-19 has infected a lot of people around the world. The healthcare systems are overwhelmed because of this virus. The intensive care unit (ICU) as a part of the healthcare sector has faced several challenges due to the poor information quality provided by current ICUs’ medical equipment management. IoT has raised the ability for vital data transfer in the healthcare sector of the new century. However, most of the existing paradigms have adopted IoT technology to track patients’ health statuses. Therefore, there is a lack of understanding on how to utilize such technology for ICUs’ medical equipment management. This paper proposes a novel IoT-based paradigm called IoT Based Paradigm for Medical Equipment Management Systems (IoT MEMS) to manage medical equipment of ICUs efficiently. It employs IoT technology to enhance the information flow between medical equipment management systems (THIS) and ICUs during the COVID-19 outbreak to ensure the highest level of transparency and fairness in reallocating medical equipment. We described in detail the theoretical and practical aspects of IoT MEMS. Adopting IoT MEMS will enhance hospital capacity and capability in mitigating COVID-19 efficiently. It will also positively influence the information quality of (THIS) and strengthen trust and transparency among the stakeholders.

## I. Introduction

In early 2020, the world experienced a deadly infectious disease caused by the coronavirus pandemic [1]. For example, as of 17^th^ April 2020, 5,251 COVID-19 cases including 86 deaths and 2,967 cases of recovery were reported by the Ministry of Health (MOH) in Malaysia. In the same period in the USA, a total of 8,861 new cases and 961 new deaths were reported. The number of cases increased exponentially in the USA as well as in Italy, the country outside China with the most COVID-19 patients until March 29, 2020, where up to 12% of all positive cases required ICU admissions [2], [3]. COVID-19 pandemic has overwhelmed the healthcare systems and medical staff in countries around the world. The systems that have faced real constraints in capacity and accessibility during normal times have worsened during the outbreak, thus leading to worse clinical outcomes [1], [4]. The peak demand for hospital facilities due to the COVID-19 pandemic is expected to surpass capacity considerably. Despite the introduction and execution of social distance initiatives, there is an immediate need to establish and adopt strategies to decrease non-COVID-19 pandemic demands and temporarily increase the capability of health facilities [5]. THIS is an integrated healthcare information system that provides a complete paperless healthcare environment based on information technology (IT) which serves to improve the data collection process, storage, and sharing of medical information between modules in the system using standard application interfaces (APIs) [6], [25]. THIS has been implemented by MOH in Malaysia in several public hospitals such as Selayang, Putrajaya, and Sungai Buloh.

An Intensive Care Unit (ICU) is a special facility within THIS that is dedicated to treating patients who are critically ill [3]. In the context of the COVID-19 pandemic, ICUs need to increase their capacity of operations due to growing population and other socioeconomic factors such as the increasing number of people moving between countries, which has influenced healthcare systems’ capacity around the world. Medical Equipment Management System (MEMS) refers to medical equipment’s asset management where the system manages medical devices within a hospital supported by an automated system [7]. The motivation behind discussing MEMS of ICUs is that the healthcare departments that provide intensive care can access advanced medical equipment not routinely available elsewhere. Therefore, the type of data and its sources are entirely different from those available in normal departments [8]–[10]. Some challenges that exist in the current MEMS need to be resolved. For example, the risk of outbreak and its relations with the shortage of medical equipment, poor coordination of intensive care teams, and lack of information required for efficient clinical and managerial decisions. In contrast, unwise decisions may have caused wastage of medical equipment in ICUs such as beds, ventilators, defibrillators, and other scarce medical equipment [8], [10], [11]. There is a need to improve ICUs by promoting data collection process’ quality in the current THIS adopted by several hospitals around the world such as Malaysia, Korea, and the USA. Also, it is necessary to review the reallocation process of scarce medical equipment to eliminate tasks that require substantial time but may be of limited value to the front-line medical teams or the patients during the pandemic [7], [12], [13]. Researchers have pointed out that IoT has a greater capacity for vital data transfers in the healthcare of the new century. The integration of IoT with medical equipment would reduce smart healthcare systems’ total expense and reaction time [24]. Today, in the medical field, IoT technology seems to provide extensive applications towards customizing different tools and devices to meet healthcare requirements to strengthen the response of hospital information systems during the pandemic [14]. Utilizing this technology to control the outbreak has proved its efficiency in Wuhan, China, where drones were used for monitoring citizens to ensure impermanent quarantine in the Wuhan city and mask utilization [12]. Also, IoT aids in increased safety in ICUs and across hospitals, as well as improving the quality of ICU healthcare services which will impact efficiency on overall hospital capabilities at a lower cost. It can provide decision makers with more accurate information when formulating healthcare policies during a pandemic outbreak by developing MEMS in hospitals [15]. IoT is an automated solution used for data transfer, analytics, and storage collected by sensors incorporated in ICUs’ medical equipment. The information generated will be shared across diverse platforms and applications which can be used for analytics and decision making through a central server of a hospital [14], [16]–[18]. Radio Frequency Identification (RFID) is one of IoT’s automated data collection technology. An RFID reader is installed in medical equipment (bedside device) by adopting appropriate communication standards such as Medical Information Bus (MIS) or Message Queuing Telemetry Transport (MQTT) to connect with other devices such as web server in the healthcare institution and by using proper communication protocols such as TCP/IP via Internet terminal. The RFID readers are placed in the bedside devices in ICUs to identify, track, and monitor the medical equipment that is tagged automatically and in real-time. RFID can also assist in inventory management and patient location identification [19]–[21]. In this paper, the authors choose the Selayang Hospital located in Selayang in the Gombak District, Selangor, Malaysia as the case study. The hospital is a government institution with a capacity of 960 in-patient beds and 20 clinical disciplines. The hospital has adopted the THIS model to achieve its main objectives which are providing high-quality healthcare services, becoming an effective organization, and meeting the world standards [22], [23].

In this paper, we propose an IoT-based paradigm to empower the MEMS of ICUs in light of the COVID-19 pandemic. This proposed paradigm guarantees the highest level of transparency and fairness during the outbreak in the redistribution of medical equipment in ICUs. This is different from existing works that have mostly focused on adopting IoT technology in patients’ health monitoring. IoT MEMS consists of a theoretical framework for main dimensions of ICUs that are correlated with the COVID-19 pandemic and their interrelationships. It is a practical model that presents how the theoretical framework aspects can be integrated with the current THIS. The outcome of this study will assist healthcare decision-makers to restructure ICU information systems to improve the information quality efficiency of THIS. Hence, achieving the highest level of transparency and fairness in ICU medical equipment reallocation during the outbreak. The study therefore will answer the following question “how to design an IoT-based theoretical and practical paradigm for managing ICU resources efficiently during covid-19 pandemic outbreak?”. the rest of the paper is ordered starting from section ii that presents related work. section iii presents the study design of this model in Malaysia. section V discusses the proposed IoT MEMS dimensions while section v presents the research method. section vi discusses the proposed IoT mems and section vii contains the discussion. finally, section viii outlines the conclusion and future work.

## II. Related Work

Resource limitation, infection control, and protection of ICU medical staff at the front-line are the most known challenges throughout the outbreak of the COVID-19 pandemic. Goh *et al.* [25] reviewed the essential principles and strategies of ICU preparedness. The study concluded that it is important to ensure adequate supplies in ICUs during the outbreak to maintain continuous healthcare services. IoT is an innovative technology used from the fourth industrial revolution (IR 4.0) which connects biosensors and other medical devices via healthcare applications to collect, record, transfer, and share data to enhance communication between stakeholders. It also supports decision-makers to take proper decisions within a shorter timeframe during the outbreak of the COVID-19 pandemic [16], [17], [26].

**TABLE 1:** Comparisons Table of IoT Based Health Care Management System

Paper Title	Objectives	Integrated with THIS	Process Flow	Outcome	Gap	Ref
IoT-Based Information System for healthcare application	Activity Recognition	N	Y	Fall detection and behavior recognition and classification	Need verification	
IoT-based intensive care secure framework for patient monitoring and tracking	Patient Health Tracking	N	Y	Prototype exists	Need verification	[37]
Smart technologies drove approaches to tackle COVID-19 pandemic: a review	Review Paper	N/A	N/A	N/A	N/A	[28]
Role of IoT to avoid spreading of COVID-19	COVID19 Spearing Mentoring	N	Y	Conceptual model	Need verification	[29]
IoT Based Health Care System Pandemic (Covid-19) Aspirant Using Cloud Computing	Healthcare Personalizing	N	Y	Conceptual model	Need verification	[38]
Monitoring System IOT Based ICU Patient Health	Patient Health Tracking	N	Y	Prototype	Need verification	
Internet of things for healthcare monitoring applications based on RFID clustering scheme	Patient Health Tracking	N	N	Simulation	Need verification	
A framework of medical equipment management system for the in-house clinical engineering department	Medical Equipment Management	N	Y	Currently used	No IoT components	[7]
SHPI: Smart Healthcare System for Patients in ICU using IoT		N	Y	Combine IoT and blockchain Healthcare System	Need verification	[7]
Smart and Pervasive ICU Based-IoT for Improving Intensive Health Care	Patient Health Tracking	N	N	Conceptual model	Need verification	
IoT-Based Health Monitoring System for Active and Assisted Living		N	Y	Prototype	Need verification	
IoT-Based Remote Pain Monitoring System: From Device to Cloud Platform	Patient Health Tracking	N	Y	Prototype	Need verification	
Real-Time Patient Monitoring System based on Internet of Things	Patient Health Tracking	N	Y	Prototype	Need verification	
Secure IoT-Fog Enabled Smart Decision-Making system using Machine Learning for Intensive Care unit	Health Care Decision Support System	N	Y	Prototype	Need verification	[39]
Medical equipment management system design method based on intelligent Internet of things	Medical Equipment Management	N	Y	Prototype	–	[34]
Management platform of medical testing equipment and testing data, and Internet-of-things application method	Medical Equipment Management	N	Y	Prototype	–	[36]
Medical equipment management system based on Internet of Things	Medical Equipment Management	N	Y	Prototype	–	[35]

IoT has added a new value to ICUs by offering novel methods, architectures, and solutions for enhancing their capabilities and capacity in fighting the novel coronavirus outbreak. IoT combined with other technologies such as cloud computing, artificial intelligence, and machine learning is one of the best-utilized innovations in combating the COVID-19 pandemic in healthcare institutions in general and ICU departments in particular. IoT provides the ICUs with proper services to control COVID-19 by using remote health monitoring systems (telemedicine services) and biosensors to provide useful information to patients and doctors [16], [17], [26].

Dziak *et al.* in an article entitled “IoT-Based Information System for healthcare application” released in 2017 stated that one of the most significant issues in IoT healthcare applications is the localization of patients or equipment. Another study by Omran *et al.* released in which proposed an IoT-based intensive care secure paradigm for patient monitoring and tracking mentioned that IoT can provide smart health systems to enhance working efficiency while considering service quality in ICUs. Another study by Khan *et al.* which was published in 2020 proposed a smart helmet integrated with IoT technology for monitoring infected COVID-19 patients using facial-recognition and thermal camera technology to get real time data [28]. Kumar *et al.* in a study entitled “Role of IoT to avoid spreading of COVID-19 pandemic” pointed out that IoT applications in healthcare are helpful to improve access to patient information using cloud computing (CC). Based on the data collected in real-time by the ICU devices, healthcare experts can do statistical analysis to make decisions during the pandemic [29]. A research paper by Chaudhari *et al.* released in 2020 discussed the role of IoT during the pandemic and concluded that a better integration environment in hospitals can be implemented by using IoT applications [31]. Another research by K. Kushwaha discussed IoT-based health care systems during pandemic using cloud computing [32]. They pointed out that hospital information systems can be designed based on IoT to improve communication channels. This serves to support front-line physicians in critical care to monitor COVID-19 patients’ conditions using biosensor nodes to collect more precise information about the pandemic. Dudhanikar *et al.* in an article published in entitled “IoT based ICU monitoring system” concluded that by adopting IoT-based systems in ICUs, patients are well-monitored through continuous tracking of their health information using biosensors and medical devices. A study by Abuelkhail *et al.* entitled “Internet of things for healthcare monitoring applications based on RFID clustering scheme” discussed developing a paradigm for IoT based on a clustering scheme involving RFID integration with wireless sensor systems to collect information efficiently. The smart nodes in the proposed clustering are composed of RFID tags to collect data to be sent to the RFID reader. This leads to a proposed paradigm that can effectively monitor people’s health at the time of the pandemic outbreak.

In the context of MEMS, a study by Chien *et al.* [7] addressed medical equipment management systems and proposed a paradigm for in-house clinical engineering departments. The study concluded that a medical equipment management system can improve the quality of information which can advance the quality of operations. Also, it can reduce the cost of maintenance, as well as boosting medical devices’ safety which is used by patients and medical staff in healthcare facilities. The study added that by using an efficient medical equipment management system, healthcare facilities will be able to face potential challenges. M. S. Kumar *et al.* [33] who conducted a research under the title “Applications of industry 4.0 to overcome the COVID-19 pandemic operational challenges” pointed out that due to COVID-19, the demand for medical equipment and accessories has significantly increased. Therefore, there is a need to enhance transparency and trust in managing medical equipment. Using information technology to handle the lack of necessary medical equipment during the COVID-19 pandemic in medical facilities is considered one promising area of research. Utilizing IoT-based ICUs during the time of COVID-19 is still in its preliminary stage. Cao Xiaowu *et al.* [34] designed a technique to manage medical equipment based on intelligent IoT. Specifically, the process consists of the following steps: S1, inputting equipment information and producing an electronic tag; S2, setting up equipment borrowing procedures; S3, equipment tracking; S4, returning, informing, and viewing equipment. Dai Yueyue *et al.* [35] proposed a medical equipment management system based on IoT. They focused on managing the maintenance time of each medical system and avoiding excessive errors caused by missing the optimum maintenance time. Chen Weiyu *et al.* [36] proposed a management platform of medical testing equipment and testing data, and an IoT application method. The management platform comprises development, examination, and pathological testing equipment distributed through each medical section workplace, a testing data acquisition management system, a testing database AI cloud and a testing equipment management system.

Most current studies have focused on using IoT technology either in patients’ health tracking [27], [32], [38], decision support systems [29] or survey works that present its role in pandemic situations [28], [31]. Some works have been done in Medical Equipment Management field using IoT technology [34]–[36].

Studies on how to utilize IoT technology in ICUs’ medical equipment management taking into consideration the current health information system architecture like THIS are limited in number. Also, the literature that discussed this issue from different aspects including technical and management views has not provided a clear view of the best paradigm that can be adopted in case of an epidemic outbreak. To take advantage of IoT, integration with existing information systems is needed to enhance the performance and efficiency of the healthcare tasks, as well as the cost and quality of services in healthcare institutions. Therefore, this study theoretically and practically described how MEMS module can be integrated with Selayang Hospital’s THIS model in Selangor, Malaysia. The hospital still uses the barcode method to track and monitor hospital tasks such as patient status, medical inventory, and medical assets. The current THIS system does not consist of any module that manages and tracks medical equipment position, status, and maintenance in real-time. MEMS module can improve the performance of healthcare information systems (HIS) and medical services’ quality in the ICUs. It can also improve the ability of ICUs to face potential risks such as epidemic outbreaks by providing the necessary information anywhere in real-time to the front-line medical staff and decision-makers in healthcare facilities. Accordingly, the main hypothesis in this paper is that the authors presumed that to promote ICU management and the quality of service in light of the COVID-19 pandemic outbreak, the healthcare providers have to improve THIS’ information quality by adding a new MEMS component to monitor and reallocate scarce medical devices fairly and transparently.

## III. THIS

THIS is one of MOH projects in Malaysia, categorized under THIS, Intermediate Hospital Information System (IHIS), and Basic Hospital Information System (BHIS). The system has been implemented in some public hospitals such as Selayang Hospital in Selangor to improve the quality of health services to the public in Malaysia. The system was developed by Cerner Corporation, an American supplier of health information technology services and medical devices. THIS is a computer-based information system to manage all hospital processes. Its main objective is to transform the healthcare system in Selayang Hospital from a paper-based system to a paperless environment to enhance health services delivery to citizens on time, in a proper place, and an efficient manner. THIS is an integration of clinical, administrative, and financial systems that provides efficient automation of the work environment (registration, diagnostics, outpatient clinics, and billing) with the ability to store, retrieve, and update data as well as sharing information to support medical statistics for research purposes [39] [41]. The significant reason for the choice of this model in this study was the number of applications that make up this model. Also, THIS has been implemented in other hospitals such as Snuh University Hospital in Korea. The same healthcare information system has been developed by LDS Hospital University of Utah, Salt Lake City [42].

**FIGURE 1.**
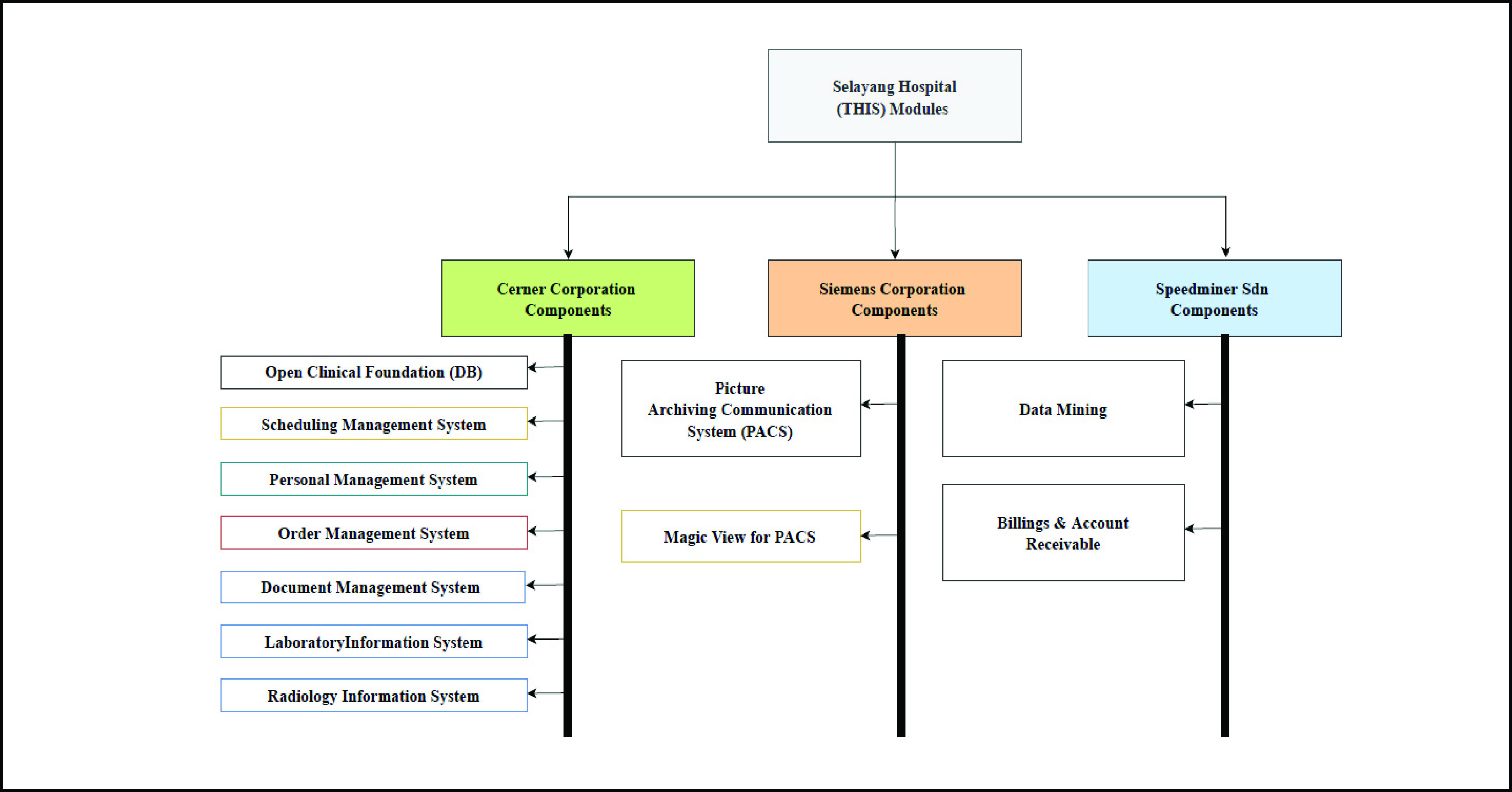
This medical devices’ suppliers.

THIS module encompasses Electronic Medical Records (EMR) as the core healthcare information system supported by Scheduling Management System (SMS), Order Management System (OMS), Administration system, and PowerChart components, integrated with Pharmacy Information System (PIS), Laboratory Information System (LIS), Expert System, Radiology Information System (RIS), and Picture Archiving Communication System (PACS). All of these components make up the core healthcare information system of Selayang Hospital. Other modules to process non-medical data such as Human Resource Management, Administration Financial System, and Material Management support systems are also integrated with the core healthcare information system to make up the THIS [39]–[41]. Fig. 1 shows the medical devices’ suppliers for THIS’ services at Selayang Hospital, which consists of three companies, Cerner, Siemens, and Speedminer. THIS as a healthcare information system has been adopted by Selayang Hospital to successfully ensure the transformation of traditional hospital activities to digital health to improve the quality of healthcare services and operations, as well as controlling the cost of services.

### A. This Subsystems

**FIGURE 2.**
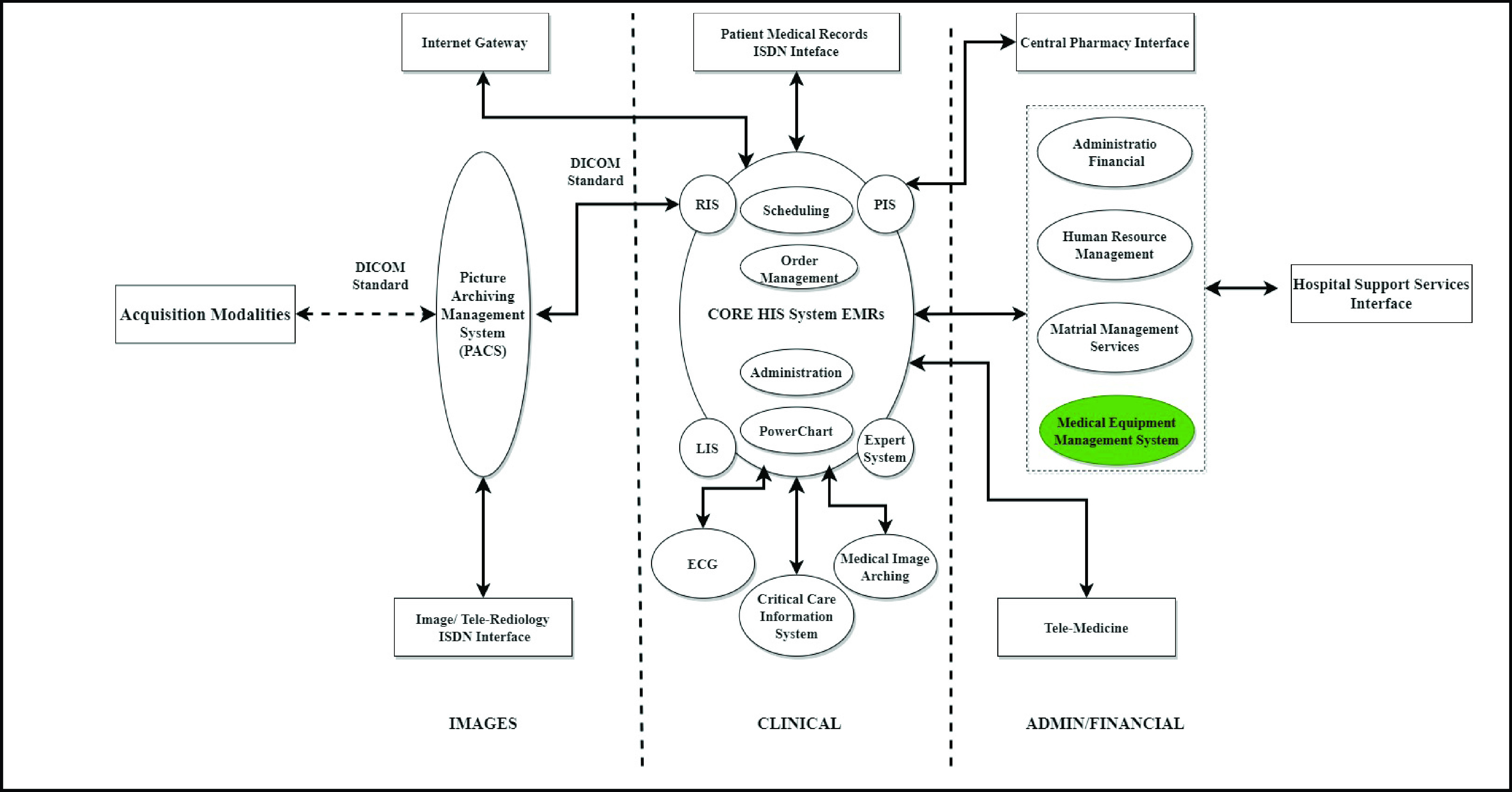
Total hospital information systems.

In the context of the THIS model, Fig. 2 shows the Patient Medical Records (PMR) integrated with other modules using ISDN interface as follows:

#### 1) Scheduling Management System (SMS)

manages outpatient clinic by giving patients proper appointments based on hospital schedules. The system can speed up treatment tasks at the Selayang Hospital’s outpatient clinic. Also, it can decrease waiting time and crowdedness in the hospital, especially during the COVID-19 pandemic.

#### 2) Order Management System (OMS)

is an electronic system that manages and monitors the orders between modules efficiently through entry prioritization by controlling the healthcare information system modules. For example, the system may be integrated with PIS or RIS to streamline medical operations, which are originally very complex.

#### 3) Laboratory Information System (LIS)

is based on a client-server system that manages laboratory tasks such as clinical chemistry, hematology, and microbiology. It is integrated with other modules such as EMR and FIS using ISDN interface.

#### 4) Pharmacy Information System (PIS)

is a computerized system responsible for managing pharmacy inventory and purchasing orders as well as physician orders using ISDN interface. It is integrated with computerized physician order entry (CPOE) that manages description orders.

#### 5) Administrative Information System

handles admission, discharge, and transfer (ADT) of patients as well as billing and accounting systems using ISDN interface for integration with other modules in the THIS model.

#### 6) Radiology Information System (RIS)

is a computer system that carries out radiology department tasks. It is integrated with Picture Archiving and Communication Systems (PACS) using Digital Imaging and Communications provided by Siemens corporation using DICOM standard for digital image format and file structure. It also displays radiology reports on PACS workstations and records the reports entered on display stations on the RIS database. Magic View for PACS can distribute images and support access to the central database in the THIS system using medical image archiving. Also, it can provide remote access to LIS service by using the Image/Tele-Radiology component with ISDN interface. Electronic Medical Records (EMR) is a computer system that can improve information sharing between hospitals using ISDN interface capabilities to support data transfers between modules. This system supports nursing activities through the nursing information system component, which is one of EMR systems that include CPOE and ADT. Patients’ documents can be displayed using the PowerChart component where it can provide access to lab results. Patient documentation can enhance decision-makers and physicians’ ability to review patients’ clinical data anywhere at any time to make proper decisions. Cerner PowerChart can also support viewing medical records and everything in charts efficiently and streamline clinical workflows. The expert system is an artificial intelligence system designed to simulate and solve complex problems and decision-making abilities. The system was provided by Speedminer. It is a Malaysian company specializing in data mining and data analytics.

Critical Care Information System is a significant system in EMR that has a great potential in enhancing critical care delivery in ICUs. The system uses HL7 standard to integrate with EMR and other healthcare applications.

Electrocardiogram (ECG) refers to the heart monitor that can check and record electrical signals from a patient’s heart based on IoT technology and artificial intelligence system using the network interface. It provides a proper channel for interconnecting systems based on Medical Information Bus (MIB), the IEEE 1073 standard designed to solve communication problems between medical devices from different suppliers using a common interface. Billing and account receivables or Financial Information System (FIS) is a computer system that manages business processes in THIS where the system integrates with other systems using ISDN interface. The Human Resources Management system manages all medical and non-medical staff affairs encompassing their duties in the hospital, their financial matters, and retirement, as well as their insurance policies.

Material Management System refers to the computer system that tracks patient information and inventory based on barcodes. Tele-Medicine and Tele-Radiology are systems that support remote access for authorized healthcare stakeholders.

### B. THIS Interfaces

#### 1) HIS Interface

It is based on order communication system (OCS), a client-server system developed to provide order entry and result reporting services to the system users. Currently, there is only one channel for sending data to OCS. The types of data transferred include admission, transfer, discharge, and scheduling, along with patient demographics using TCP/IP socket.

#### 2) Network Interface

This is the lower level of the system integration paradigm. HIS is connected to the hospital LAN with a special gateway unit (MTX 9430) that provides a channel interface to the host.

#### 3) Database Interface

It is the middle level of the system integration model supported withOpen Clinical Foundation (OCF) which refers to a relational database. It is reinforced with multimedia capabilities that can gather and process data produced by core systems to form electronic medical records as well as supporting data extraction for research purposes. The database interface is in the mid-level of system integration to support data exchange between THIS components. The most important feature of this database is its failover capabilit.

#### 4) Workstation Integration

This is top-level in the THIS integration model that supports end-users with a standard interface to access all medical information systems such as PACS, RIS, LIS, and EMR [25], [22], [39], [41], [43].

Although the amount of data recorded for ICU patients increases every year, most ICUs in hospitals and particularly in developing countries use paper-based medical records rather than digital records for their clinical practices. However, over the past few years, ICUs in several hospitals around the world have experienced the advantages of healthcare digitalization such as North America, Canada, and Europe by utilizing smart Critical Care Information Systems (CCIS) [44]. The ICU environment is multi-disciplinary. This unit depends on teamwork and a set of healthcare applications such as CCIS that integrate with other systems in HIS that include CPOE, Clinical Decision Support (CDS), EMR, and PACS to achieve tasks. Furthermore, THIS involves a group of networking technology, medical databases, electronic medical records, and CCIS. The roles of these modules in ICUs include collecting, processing, and presenting data for use in patient care [44]–[46]. In the context of benefits, CCIS improves the efficiency of ICU processes and tasks, and clinical data structure, and during the outbreak [44], [46], [47]. ensures data availability for ICU medical staff [44]. Ever since the COVID-19 outbreak, healthcare systems are committed to rapid responses through advanced technology to meet the needs of healthcare providers that have received many requests for tracking surges, COVID-19 pandemic positive tests, bed capacity, and other scarce medical equipment, especially in the ICUs. Healthcare providers with their partners such as Siemens and Speedminer are working together to empower healthcare systems through Artificial Intelligence (AI) and IoT to ease the burden of front-liners to control the outbreak. To summarize, an ICU consists of a set of resources, devices, and clinical practices to support medical staff to achieve the healthcare organization’s goals in terms of the quality of healthcare services. Poor system usability such as difficulty to access certain information and wrong document capturing can impact the ICU process flow and the outcome. Also, system failure can affect the information flow which will adversely impact the quality of ICU services [44], [47]. To improve the preparedness of ICUs during the COVID-19 pandemic outbreak, it is crucial to improve the quality of the information in clinical information systems by using real-time technology such as IoT for collecting medical equipment data including the location of medical equipment and medical devices’ maintenance records. This serves to rapidly guide patients based on their health conditions to the nearest ICUs to resuscitate them and avoid wasting scarce medical equipment by encouraging rationale and transparent management of such medical equipment and resources.

## IV. IoT MEMS

**FIGURE 3.**
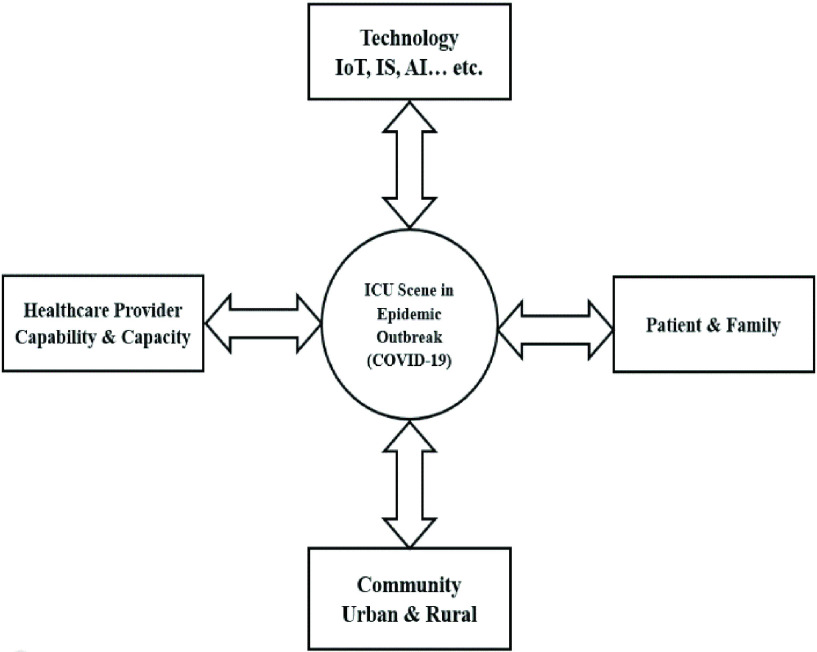
Conceptual framework of ICUs dimensions.

COVID-19 has strained healthcare systems around the world. This pandemic has extremely affected medical staff efforts in the front-line due to the shortage of medical equipment in ICUs caused by inadequate information. Also, poor communication in the current information system that complicates patients’ data exchange among physicians, departments, and even patients themselves has negatively affected the efforts in controlling the outbreak [14]. Apart from that, the delivery of critical care has its own specific needs and vulnerabilities. Although each emergency medical service is unique (type of disaster, patient casualty size, current critical care capabilities, duration of casualty-generating circumstance), they commonly encounter challenges for all mass casualty events (MCEs). Hence, all ICUs must have a general emergency medical services equipment plan in place that defines “realistic hospital capacity”, which is the admitting capacity as determined by available medical/surgical resources and equipment [48]. This study proposed a new conceptual paradigm for the information flow in ICU during the COVID-19 pandemic outbreak. Fig. 3 shows the four components as follows:

### A. Healthcare Provider Capacity

A healthcare provider’s capacity refers to the number of medical resources in ICU such as staff, space, and medical supplies (pharmaceuticals and equipment) available, while a healthcare provider’s capability refers to the quality of these resources in delivering services in ICU including the types of healthcare professionals available to render appropriate care for the patients and the quality of medical resources available to administer certain treatments for patients at the proper time and place. Therefore, a healthcare provider’s capacity and capability are considered a part of the ICU preparedness plan to improve the healthcare services’ quality [49]–[51].

### B. Community (Urban and Rural)

All ICUs are not the same. However, the growing demand during a pandemic outbreak on ICU services has overwhelmed the capability and capacity of healthcare providers in urban and rural communities. This is one of the biggest challenges faced by rural hospitals. For example, limited medical resources (ICU equipment, ICU space, number of ICU beds, and healthcare professionals) where ICU needs are completely different between urban and rural hospitals can impact the quality of patient data and medical decision-makers as well as the ICU plan. Therefore, the need to implement different strategies depending on local requirements and resources available for healthcare providers and ICUs is significant to control the pandemic. Similarly, technology, public policies, and financing models promoting the quality of services across ICUs impact the level of discrepancies between urban and rural healthcare providers. Therefore, preparing for future surges of a pandemic is imperative to increase ICU capability and capacity, and manage complex cases in rural hospitals [52]–[55].

### C. Patient and Family

The patient and family need to play their roles in supporting the front-line medical staff, where their awaraness of ICU tasks is the most important component to support the ICU preparedness plan to curb the COVID-19 pandemic. For example, healthcare providers must encourage family members to be a part of the team during rounds, procedures, and bedside care because they can play a critical role in patient safety in the ICU, as well as reducing the period of patient occupancy on ICU beds. Also, communication and transparency are improved when family members are a part of ICU care. Thus, the ability of family and patient to use technology such as ICU applications and healthcare wearable devices can enhance communication among the ICU team, the family, and the patient [51], [56], [57].

### D. Technology

A multitude of techniques has been instituted in ICUs by providers to support patients’ needs. Therefore, technology has the capacity to enhance the quality of healthcare services in ICUs by providing the best solutions to front-line clinicians and auxiliary staff to control the pandemic. Digital technology can be implemented to enhance speedy response to demands of ICU services, where it can guide patients and medical staff to take proper decisions. For example, technology can decrease the rate of mortality due to limited resources by improving communication between ICU medical staff in different hospitals as well as between patients and clinicians. Also, technology can increase trust and transparency between healthcare stakeholders during the pandemic where it can assist healthcare decision-makers to control the spread. Digital healthcare speeds up the transformation of telemedicine consultation especially during an outbreak to control patients from moving and spreading the virus. Furthermore, IoT, AI, big data, cloud computing, autonomous robots, and biosensors can play a significant role to speed up the response of ICU preparedness. Therefore, basic information technology architecture is needed in ICUs to support TEL-ICU applications shortly to enhance the adoption of digital health [16], [26], [53], [58]. An ICU must be able to function independently by using the latest technology such as IoT to overcome obstacles and challenges in fighting future pandemics [30]. For example, using RFID tags or Bluetooth to detect ICU medical equipment and access up-to-date operational or maintenance records. These facilitate more informed decisions, either to guide patients with critical conditions to the nearest ICU or manage available ICU equipment with more efficiency and transparency to save people’s lives [8], [48], [59]. Fig. 3 shows the proposed conceptual framework of ICU components during COVID-19.

## V. Research Setting

The appropriate methodology for this research paper was qualitative research based on a narrative inquiry approach, aimed to answer the research question. Thus, the information gathering and paradigm development in this article utilized existing knowledge on this topic to support a methodology to improve the capabilities of current THIS in the Selayang Hospital’s ICU during the COVID-19 outbreak. However, research on IoT as one of the Industry 4.0 components in the medical field is an upcoming venture. Data was extracted from digital libraries by using keywords such as “IoT”, “internet of things”, “medical internet of things”, “total hospital information systems”, “Intensive care unit”, “Critical care unit”, “IoT-base healthcare paradigm”, “Medical equipment”, and “Coronavirus (COVID-19 pandemic)”. To achieve the research aim, the authors structured the research methodology by collecting information from secondary sources such as scientific articles, specialist newspapers, databases, and websites, as well as related information released by government departments, management agencies, medical institutions, healthcare industry associations, and public enterprises. These served to understand the ICU information flow and data sharing actions with other departments in the same hospital or with other healthcare institutions in the country. The main scope of the explorative phase was to collect information on ICU process flow, THIS model, and IoT in ICUs and the healthcare field. In particular, the authors first studied the topic and undertook a review of existing literature on the main topics. Ultimately, the authors analyzed all the data gathered with details on IoT-based ICUs related to THIS requirements and research question which served as a structure to assess each element in the IoT-based paradigm to upscale the THIS performance in ICUs in light of the COVID-19 pandemic.

## VI. The Proposed IoT MEMS

**FIGURE 4.**
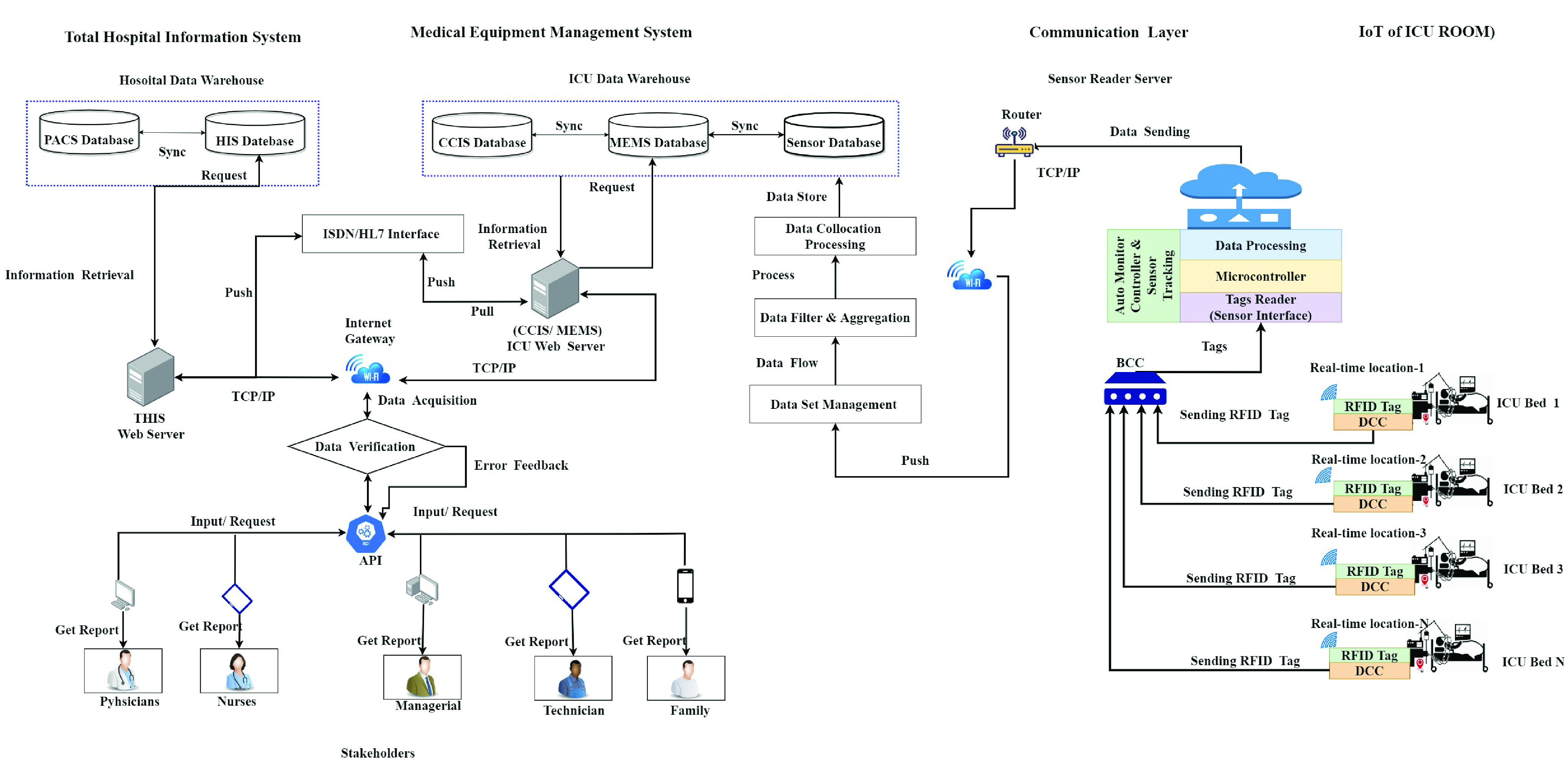
Proposed MEMS integrated with THIS.

This paper lays out the THIS model supported by the new proposed component, IoT MEMS as shown in Fig. 4. IoT MEMS handles the reallocation problem of scarce medical equipment including ICU beds, ventilators, and defibrillators during the COVID-19 pandemic. Also, it will scale up the ICU performance as well as improving the quality of information needed by the decision-makers and front-line medical staff. The proposed IoT MEMS components are as follows:

### A. Essential Components

#### 1) Hardware

It is the tangible component of any information system that consists of input and output devices and a central processing unit used to capture data and present it to the stakeholders. The hardware in ICU is divided into three groups: 1) Servers to store and process data, and host various applications (web server, database server, and sensor reader server), personal computers, and peripherals such as scanner and printer; 2) Things and devices in ICUs that include beds and medical equipment such as ECG monitor, ventilator, and wearable devices; and 3) Various network equipment and tools that include cables to link RFID components, routers, and access points.

#### 2) Software

It is a set of instructions or data to direct the devices (computer, sensors, and medical equipment) to execute a specific task to collect, process, and output information for use. By identifying the software components’ capacities and enhancing the compatibility between them, it will be able to transfer and exchange medical information across subsystems within the integrated healthcare information system successfully.

There are two main types of software in the proposed paradigm: 1) The operating system to provide the platform and user environment for ICU applications, where the common systems are UNIX-based systems, Microsoft Windows, and ISO or Android for handphones; and 2) The applications where they can be integrated with MEMS such as CCIS that include other healthcare information system components in THIS. For example, CPOE sends medication orders and treatment recommendations via a computer application as a paper replacement and ADT improves patient care coordination within the same hospital or with other hospitals. Other important applications include LIS, PIS, and PACS where the medical technology is used to store medical electronic images in the proper format.

#### 3) Communication Layer

It is the backbone of IoT system. It is regarded as the mid-layer between the application layer and IoT system activities. The communication channels in the IoT system can be wireless or wired based on the network design and selection of the proper protocol. The proposed IoT system needs to have a smart network infrastructure to overcome connectivity problems that can cause the inability to access any network resource. Also, connectivity is a primary user requirement that can support data exchange between people and IT devices, and increase network reliability and availability. Network connectivity empowers IT devices’ performance and improves communication among each other. The importance of connectivity increases dramatically across the world with the demand increase in IoT technology usage in different industries. Fig. 4 shows that all the targeted ICU medical equipment will install RFID components to connect with other IT devices and share the required information. Besides, the required information including equipment ID, real-time location, type, state, and maintenance report will be encapsulated and sent to the proposed MEMS system by using RFID tags. It will be updated using real-time analytics. The data is obtained by IoT sensors embedded in the medical equipment and devices in the ICU and transmitted over the Internet via network components to the IoT application. Another important thing that should be considered in communication layer design is standardization. It is one of the obstacles that hinder the implementation of automated data collection in medical equipment and the health domain, while other industries have benefited from standardization in their domains. For example, HL7 is the standard for hospital admission/discharge/transfer and order/result transactions; Digital Imaging and Communications in Medicine (DICOM) for radiological imaging; and Healthcare Informatics Standards Board (HISB). A major contribution is concerning healthcare insurance portability and accountability. MIB is used to identify the connectivity standards between ICU devices such as bedside devices (infusion pumps, ventilators, defibrillators, and oximeters). All of these health standards are approved by the American National Standards Institute (ANSI). The proposed communication standard to use with this system is MIB standard IEEE 1037, which is the lower layer standard. Fig. 4 shows that a bedside hub configuration consists of devices including infusion pumps, defibrillators, ventilator, and oximeter. This hub comprises the Bedside Communication Controller (BCC) where each device exchanges data through Device Communication Controller (DCC), either internal or external. A clinician can plug in any bedside device from any manufacturer into the MIB-BCC port without any technical intervention.

#### 4) Human

Human, people, or stakeholders may refer to an individual, a group of individuals, or organizations that have a connection to the healthcare information system as users. The stakeholders in this ICU scenario are usually healthcare professionals (doctors, nurses, attendants), technicians, pharmacists, lab technicians, healthcare authorities such as government authorities (e.g. auditing), insurance companies responsible for validating and authorizing these health information processes, and those who need to access healthcare data under specific conditions.

#### 5) Data & Information

Data is the raw material collected by MEMS using RFID sensors from medical equipment in ICUs for processing and analytics, while information is an organized, contextual data useful to humans, presented as outcomes of MEMS. The proposed framework was designed to manage various ICU data such as ICU equipment data and patients’ clinical data. The collected data has a direct effect on the quality of ICU services. The data and information components refer to the infrastructure, resources, processes, and mechanisms needed to leverage informatics to achieve the goals of healthcare facilities.

#### 6) Process Flow

Two types of processes/practices are information system processes/practices (information technology process) and organizational or business processes/practices (clinical and non-clinical processes). One of the vital roles of information systems is to support business processes. Information system processes include collecting, filtering, processing, creating, and sharing data with other subsystems. The business processes or business functions point out a set of structured tasks or procedures at the organizational level that is suitable for the organization’s capabilities and meet its business goals to improve customer satisfaction and support the ability to absorb rapid market change. The assessment of organizational processes is still essential to secure better healthcare services quality and continuous improvement of the healthcare information system. Fig. 4 outlines the process flow of the proposed IoT-based system of an automated MEMS in ICU. The diagram illustrates the way to improve the quality of extracted data from patients and medical equipment. The activity diagram of the proposed system is divided into three stages which are input, process, and output data. These three stages are responsible for the improvement of data aggregation quality in CCIS. Two types of input data in the proposed system for ICUs are business data and medical data. Business data consists of admission, discharge, and transfer data as well as payment data, equipment maintenance report data, and medical equipment operation guidelines. Meanwhile, medical data comes from different systems such as LIS, CPOE, and PACS. This data can also be extracted from biomedical sensors.

#### 7) Input

The framework in Fig. 4 outlines the data sources in CCIS and MEMS systems. They come from three types of inputs: 1) Manual input which is usually entered by users (medical and non-medical staff); 2) Automated entry or data sharing where the required data is exchanged with other components in THIS such as EMR, LIS, PACS, and PIS; 3) Data sources produced by IoT-based medical equipment (ICU devices) using RFID tags that match the medical equipment and send data to MEMS or biosensors on wearable devices that monitor patients’ status to send the obtained data to CCIS. In other words, data produced from IoT-based Automated Medical Equipment Management System in an ICU either by ICU medical staff’s notes using CCIS or from the ICU medical equipment using RFID tags and patients’ wearable devices, as well as data coming from other systems. Recently, automated data collection has become an urgent matter, because it ensures the improvement of ICU system performance and quality of outcomes. The system can reduce errors and staff workload as well as avoiding the waste of scarce medical resources, and thus remedy service costs at the time of the pandemic.

#### 8) Data Processing

Fig. 4 demonstrates the process flow of the proposed IoT-based automated MEMS in ICUs. Data processing is a very important stage in ICU systems because it is responsible for the quality of data collection and aggregation as well as the type of output information in the proposed system. Information is needed by clinicians and decision-makers to improve ICU performance during the COVID-19 pandemic. Hence, the significance of information depends on the quality of data processing before being presented to the stakeholders. There are two types of data processing which are simple process and complex process. The simple process is a normal data processing to identify values and perform simple measurements for patients in CCIS such as hypotension and temperature. It can represent a simple value to MEMS such as attitude towards ICU bedside equipment and maintenance history for ICU medical equipment. The complex process consists of very complex algorithms using machine learning methods and the data is presented to the stakeholders in the proper format to support them in their ICU tasks. Specifically, complex data processing can support clinical decision-making during critical times by integrating with other information systems such as expert systems, PACS, and EMR. In the context of data processing, the diagram in Fig. 4 outlines the data processing flow that consists of three main activities: data set management, data validation, and data collection processing. As a result, the ability to process medical equipment data, for example, bed locations, ventilators’ machine maintenance reports, and patient’s medical information in real-time helps ICU professionals to scale ICU performance together with the quality of services.

#### 9) Output

Fig. 4 shows that the information is produced by hardware or other systems and pushed to CCIS or MEMS for processing and storing in the ICU database. Later, this stored data is pulled by other computer systems such as EMR, CCIS, MEMS, and CPOE in THIS, other medical devices, or internal and external users such as ICU clinicians and nurses, and even patients, based on specific requirements. The output information in MEMS is either by user request from the proposed system (pull) or the MEMS system sending information to other systems and users that include alerts or warning messages (push).

### B. Security and Privacy Aspects

In the context of IoT MEMS, some security and privacy concerns need to be addressed. For example, node resource limitations of IoT devices do not support the implementation of more advanced security and privacy techniques. Therefore, we suggest adopting the security framework suggested in [24] to secure the data transaction of the IoT MEMS. Restricted Application Protocol (CoAP) [61] and Datagram Transport Layer Protection (DTLS) [62] protocols can also be used to ensure data confidentiality between IoT devices and THIS system. Regarding patients’ privacy, we recommend adopting blockchain to secure patients’ data as suggested in several types of research [63]–[66]. Another issue is the mitigation of potential security issues between the different information systems that are contacted through interfaces. It is suggested to use blockchain technology to validate data integrity between the different data stations on MEMS. Some works have suggested a similar solution as in Anwesha Banerjee *et al.* ICU webserver can be vulnerable to attacks such as Cross-Site Scripting (XSS), phishing, SQL Injection (SQLi), Path Traversal, Distributed Denial of Service (DDoS), and data leakage. In this context, we suggest adopting a web application security system [67] to mitigate the front-end threats as well as getting the benefit from the solution suggested in [68]. To protect from potential IoT node attacks such as radio interference and physical tampering, it is recommended to employ the deep learning method suggested in [69]. In addition, as HL7 protocol security is vulnerable to some data breach attacks, it would be better to use Triple Data Encryption Algorithm (TDES/TDEA) suggested in [70].

### C. IoT MEMS Integration Constraints

Finally, some important constraints should be considered by healthcare stakeholders when adopting IoT technology in MEMS. Before implementing the proposed system, the project manager should obtain the required resources (IT experts, medical equipment) and an adequate budget for this project to ensure that any failures or risks relevant to the system can be reported early. The outcomes of CCIS and MEMS can be achieved as the goals, objectives, and deliverables of the ICU. The stakeholders have to monitor the system quality after the implementation to ensure that it meets the ICU requirements. To guarantee the high productivity of ICU services, there is a demand to upgrade ICU tasks for utilizing IoT technology efficiently. Moreover, to ensure a successful implementation of the proposed paradigm, the project leader should encourage contributions from all healthcare stakeholders such as top managers, IT staff, clinicians, and nurses who are in direct ICU relevance and can be considered as end-users of MEMS or CCIS during the implementation. The above implementation constraints need to be recognized early and observed to ensure the improvement of information quality in the clinical information system.

## VII. Discussion

The accelerating change in healthcare systems due to the COVID-19 pandemic has highlighted the need for health sector automation. However, access to accurate information and assurance of information integrity during data transfers to the healthcare information system from bedside devices are still considered as the real challenges. In general, the adoption of standard processes in hospitals and integration between RFID and medical devices based on IoT in HIS can enhance the automation of healthcare systems. In this study, we conducted a comparative investigation using the existing literature on the most significant healthcare information system models implemented recently in developed and developing countries such as Malaysia, Korea, and the USA. This served to understand the obstacles behind medical equipment utilization in the ICUs, which have burdened front-line medical teams during the COVID-19 pandemic around the world. These can help us to perceive the weaknesses in the current THIS model. The current THIS model as shown in Fig. 2, which has been implemented in some Malaysian hospitals includes the material management support services system. As described briefly in Section (3), this component uses the barcode method as a means of quick identification and tracking of medical materials in warehouses. The same technique is also used for tracking patients and medical assets in hospitals. Moreover, there is no component in the current THIS model for tracking medical equipment positions, current status, and even the maintenance and troubleshooting history records especially in ICUs where real-time information is important for both patients and clinicians. During the COVID-19 outbreak, several challenges have emerged relating to the medical equipment management in ICUs around the world due to the lack of medical equipment and their information which have confounded decision-makers and front-line medical teams. These may impact the quality of services in ICUs due to the wastage of scarce medical equipment. Thus, everyone has become painfully aware that severe shortages in ICU capacity and capability have negatively impacted the efforts of healthcare professionals around the world to address the pandemic.

IoT MEMS paradigm, as proposed in this paper empowers the current THIS capability to manage ICU medical equipment efficiently as shown in Fig. 4. The proposed paradigm intends to use RFID technology as one of IoT components to monitor medical equipment locations, conditions, as well as presenting reports on ICU capacity and capabilities to healthcare decision-makers and clinicians as they are not available in the current THIS. This module is added to THIS which is built on IoT technology to integrate with ICU medical devices to collect existing medical equipment data in real-time and send this data to MEMS to store in the medical equipment database to be accessed later based on stakeholder requests. It has become important to embed IoT into THIS to increase information transparency about pandemics such as COVID-19, reduce stakeholders’ anxiety, and enhance public confidence in the measures that government and healthcare providers take to combat them. Furthermore, according to previous studies, the authors will state several benefits of adding MEMS to THIS.

### A. Theoretical Contribution

The key objective of this research is to add IoT MEMS to THIS, which will enhance the system’s information quality to ensure the highest degree of accountability and justice in the reallocation of medical equipment in ICUs during the outbreak. In a theoretical context, this study has introduced a new conceptual model of the information flow between THIS and ICUs during the COVID-19 pandemic which will have a direct impact on ICU information systems’ quality. It involves four dimensions: healthcare provider capacity, community (urban and rural), patient and family, and technology. IoT MEMS section has provided more details, as seen in Fig. 3.

### B. Implications for Practice

Apart from the theoretical contribution, it provides a technical model in terms of how the conceptual models can be practically integrated with the current THIS architecture. The new proposed architecture would also enhance the productivity of ICUs and improve the information accuracy needed by decision-makers and front-line medical staff. This is achieved by feeding CCIS with more reliable inputs as seen in Fig. 4.

## VIII. Conclusion and Future Work

It becomes crucial to improve the quality of extracted information from medical equipment in ICUs to support front-line medical staff and decision-makers and ensure efficient utilization of ICUs’ capabilities and capacity during the COVID-19 outbreak. This paper introduced a modern IoT-based paradigm to successfully handle ICU medical equipment, called IoT MEMS. It utilizes IoT to improve information flow between THIS and ICU during COVID-19 to ensure the highest degree of accountability and equity in the reallocation of medical equipment. Adopting the proposed model would boost the capability and ability of hospitals to successfully reduce the spread of COVID-19. The accuracy of the information in THIS will also be significantly impacted, and confidence and accountability among the stakeholders will be improved. It would ease the access to medical devices’ information anywhere in real-time. Also, it can reduce maintenance costs and duplication of information entries. IoT MEMS consists of a theoretical and practical model to be integrated with the current THIS. This paradigm shapes a roadmap to develop new software for ICU resource management. However, some constraints need to be considered. For example, necessary services such as IT experts, medical equipment, and appropriate financial allocation must be available before implementing the IoT MEMS paradigm. Also, ensuring that any system-relevant faults or threats can be identified early. After implementation, stakeholders have to be able to track the system output to verify that it complies with the ICU requirements. Future work will involve conducting more research in the integration of AI, deep learning, and IoT with THIS, where such integration can help in improving the control of epidemic outbreaks and understanding the relevant risks with ICU tasks at the time of the outbreak, as well as improving THIS outcome.
